# UTILIZING VALID, RELIABLE, AND PRACTICAL MEASURES OF HEALTH STATUS IN PRIMARY GERIATRIC CARE: TRANSLATING RESEARCH INTO USUAL CARE WITH THE SENIOR’S HEALTH ASSESSMENT REPORT AND PLAN (SHARP™)

**DOI:** 10.1093/geroni/igad104.3691

**Published:** 2023-12-21

**Authors:** Ted Rosenberg

**Affiliations:** University of British Columbia, Victoria, British Columbia, Canada

## Abstract

**Introduction:**

To better support evidenced-based care, we combined 9 valid, reliable, and clinically useful tests into a single health status and risk assessment tool, performed by a Team nurse, called the Seniors Health Assessment, Report and Plan (SHARP™). These tests include: CFS, EQ5D-5L, EQ-VAS, MoCA, GDS, Months of the Year Backwards, Gait Speed, Grip Strength, Water Swallow Test, and MNA-6. A published study, with 18 months of follow-up for a primary geriatric home-based practice demonstrated that these tests were stronger predictors of death, nursing home transfer (NHT), or hospital admission(HA) compared to any medical diagnosis, multiple comorbidities, or polypharmacy. Hazard Ratios for SHARP™ vs medical diagnoses were: Death (median HR 5.9 vs. 1.6), NHT (median HR 4.6 vs.1.4), and HA (median HR 6.0 vs. 1.6). The research presented here will provide several case-based studies to demonstrate how we have translated this research into “usual care” for a primary home-based interdisciplinary geriatric medical practice. Specifically, we will demonstrate how we: 1. share this data with patients and caregivers to motivate them for Team interventions, 2. share a summary report of an individual’s health with the hospital and other community care providers, 3. use these tests to guide and evaluate Team interventions for individual patients (e.g. changes in gait speed), 4. Track changes in health status and risk, and 5. use aggregated data at a program level for benchmarking, defining population needs, and for planning and evaluation.

**Conclusions:**

Standardized testing is acceptable to patients, efficient, supports evidenced-based care, and is useful for program planning and evaluation.

